# Excellent long-term pain response and local control following postoperative radiotherapy in patients with multiple myeloma

**DOI:** 10.1007/s00066-024-02198-7

**Published:** 2024-01-30

**Authors:** Justus Kaufmann, Annika Ute Täubl, Eirini Nikolaidou, Alexander Rühle, Anne Hopprich, Daniel Wollschläger, Arnulf Mayer, Nils Henrik Nicolay, Heinz Schmidberger, Tilman Bostel

**Affiliations:** 1 Department of Radiation Oncology, University Medical Center Mainz, Mainz, Germany; 2 Department for Radiation Oncology, Charité-Universitätsmedizin Berlin, 13353 Berlin, Germany; 3 Department of Radiotherapy and Radiation Oncology, University Hospital Leipzig, 04103 Leipzig, Germany; 4 Institute for Medical Biostatistics, Epidemiology and Informatics, University Medical Center Mainz, Mainz, Germany; 5 Division of Radiation Oncology, Peter MacCallum Cancer Centre, Melbourne, Australia

**Keywords:** Skeletal related events, Osteolytic lesions, Epidural spine compression score, Spinal bone lesions, Spinal surgery

## Abstract

**Purpose:**

Multiple myeloma is associated with osteolytic bone lesions, often requiring surgery of the spine and postoperative radiotherapy (RT). Although common, data for clinical and informed decision-making are sparse. In this monocentric retrospective study, we aim to report the outcome of patients who underwent spinal surgery and postoperative RT due to multiple myeloma.

**Methods:**

A total of 54 patients with multiple myeloma who underwent prior spinal surgery and postoperative RT at our institution between 2009 and 2020 were analyzed. Spinal instability neoplastic score (SINS) and Bilsky score, posttherapeutic adverse events, clinical data, and outcomes were collected and analyzed. The primary endpoint of this study was overall survival (OS), secondary endpoints were progression-free survival (PFS), pain response, local control, and skeletal-related events (SRE).

**Results:**

The 3‑ and 5‑year overall survival (OS) was 74.9% (95% confidence interval [CI]: 63.5–88.4%) and 58% (95% CI: 44.5–75.6%), respectively. Median survival was not reached and 75% survival was 34.3 months (95% CI: 28.7–95.4 months). Median follow-up was 63 months (95% CI: 49–94 months). The number of patients with good to adequate performance status (Karnofsky performance score [KPS] ≥ 70) significantly increased after surgery (*p* < 0.01). We observed no grade 3/4 toxicity and only 13 (24%) grade 1/2 adverse events. Two patients (4%) experienced SRE. Overall, 92% of patients reported reduced pain after radiotherapy, with 66% reporting complete pain response. There was no difference in pain response between patients with different Bilsky scores. Bisphosphonate therapy and lower Bilsky score at the start of RT were associated with improved OS in univariate analysis (all *p* < 0.05). Multivariate Cox regression confirmed a Bilsky score of 2 or 3 as an independent negative prognostic factor (HR 3.89; 95 CI 1.4–10.7; *p* < 0.01). We observed no in-field recurrences.

**Conclusion:**

In this study, we were able to show that the current standard of RT after spinal surgery of osteolytic lesions is safe. In addition, we observed a very low rate of SRE (4%) and no in-field recurrences, demonstrating the local efficacy of RT in multiple myeloma patients. Higher Bilsky scores were associated with worse OS in multivariate analysis, but had no effect on pain response.

**Supplementary Information:**

The online version of this article (10.1007/s00066-024-02198-7) contains supplementary material, which is available to authorized users.

## Introduction

Multiple myeloma (MM) is a common malignancy with an annual incidence of 4–6 in 100,000 persons, accounting for 10% of all hematological cancers [1]. While systemic therapies including chemotherapy, corticosteroids, or autologous stem cell transplants are among the most important treatments, osteolytic bone lesions requiring some form of local therapy occur in more than 70% of patients [2–4]. Often, acute neurological symptoms ranging from pain to paraplegia require emergency surgery [5]. While bisphosphonates can increase bone stability and prolong survival of MM patients in general [6], postoperative radiotherapy (RT) in particular has been shown to improve outcomes due to a significant decrease in skeletal-related events (SRE) in patients who required surgery [7, 8]. This is likely to be facilitated by the fact that RT has a bone-stabilizing effect on such lesions [9]. Multiple studies were able to show a decrease in patient-reported pain after RT for a substantial proportion of up to 90% of patients [4, 10, 11].

A radiation dose of 30 Gy delivered in 10–15 fractions is one of the most common RT regimens in this situation and is able to achieve significant reduction of pain in most patients [4]. In palliative situations with poor prognosis, more hypofractionated treatment regimens with 20 Gy delivered in 5 fractions can be used [12, 13]. In patients with a very low performance status or low life expectancy, single-fraction RT regimens can be used. However, data regarding both oncological and symptomatic outcomes such as pain response remain sparse and inconclusive [14–16]. Furthermore, while there is a trend towards dose de-escalation, there is no consensus regarding its indication and clinical effectiveness [12, 17].

In bone metastases of solid tumors, there have been attempts to define risk factors to aid radiation oncologists in clinical decision-making with regard to radiation dose and fractionation [18]. However, while there are some data to aid in this decision-making process when it comes to patients diagnosed with MM, studies focusing on the postoperative setting are missing [19]. In addition, the increasing efficacy of systemic therapies has improved response rates and overall survival (OS) significantly [20, 21]. These improvements, combined with current demographic changes, require physicians and patients to take long-term effects on toxicity and locoregional control of these local therapies into consideration.

The aim of this retrospective study was to assess possible prognostic factors to enable allocation of patients to risk-adapted hypofractionated RT. Our data on toxicity, locoregional control, and control of symptoms after 3D-based RT should facilitate future informed decision-making.

## Methods

### Patient data

In this single-center retrospective study, a total of 59 patients with multiple myeloma who received RT after spinal surgery for osteolytic lesions between 2009 and 2020 at the University Hospital of Mainz, Germany, were identified. Five patients with solitary plasmacytoma were excluded. This retrospective study was approved by the local ethics committee (no.: 2022-16510-retrospektiv). The data selection process can be seen in Supplementary Fig. 1.

### Data collection

We performed a review of clinical records and digital images of all patients. The collected data included demographics, clinical notes, comorbidities, medication, course of the disease, RT courses, lab results, and outcome data. Symptoms were assessed mainly by review of clinical reports. In addition, if not documented, KPS was based on clinical notes, while risk scores such as the spinal neoplastic instability score (SINS) and Bilsky score were assessed by a board-certified radiologist (TB). SINS is an established risk score used to evaluate the stability of vertebrae by both clinical and morphological characteristics, dividing patients into the three risk categories “stable,” “potentially unstable,” and “unstable” [22]. The Bilsky score defines the extension of a lesion beyond the vertebral body into the epidural space [23]. Higher scores are associated with increased extraosseous extension and spinal cord compression. Additionally, other studies were able to show that the Bilsky score determines OS after spinal surgery for metastatic solid malignancies [24]. The adult comorbidity evaluation-27 (ACE-27) was calculated from clinical notes and physicians’ letters [25, 26]. The score defines four risk groups from 0 (no comorbidities) to 3 (significant comorbidities), depending on the most severe comorbidity. Additionally, if a patient has grade 2 comorbidities of two different organ systems, they are also grouped in the highest risk category. For this study, only non-myeloma malignancies were considered for calculation of the ACE-27 score. Surgical data, such as procedure and perioperative blood transfusions as well as body weight at surgery date, and postoperative complications were also recorded.

The primary outcome of this study was overall survival (OS), defined as time between the end of RT and death or last follow-up. Secondary outcomes included pain response, the incidence of skeletal-related events (SRE) and progression-free survival defined as the time between the end of RT and either death, last follow-up, or serological or radiological progression.

### Radiation treatment

In all patients, RT was planned based on planning CT (Big Bore, Philips, Amsterdam, The Netherlands) and performed by linear accelerators (Unique, Clinac or TrueBeam, all VARIAN, Siemens Healthineers, Munich, Germany) with two or more 6–18-MV photon fields. The planning target volume (PTV) included the vertebral body or bodies affected by multiple myeloma lesions and at least the adjacent disc spaces in the cranial and caudal directions. In the case of surgical stabilization by means of spondylodesis, the implanted external material was also included within the PTV. The RT regimen was chosen based on the patient’s general condition and palliative treatment goal. All patients received 3D-CRT without intensity-modulated radiotherapy. Standard treatment regimens were 30 Gy in 10 fractions. Dose de-escalation to 30 Gy in 15 fractions was done either for lesions in the cervical spine or for patients planned to receive autologous stem cell transplantation.

### Statistical analysis

Data analysis was performed using R (version 4.2.3, https://www.r-project.org/) [27]. Two-tailed Kruskal–Wallis test, Wilcoxon rank sum test, linear-by-linear association test, χ^2^ test, and McNemar’s test were used to compare differences between groups. Pairwise post-hoc Wilcoxon tests after Kruskal–Wallis tests were carried out using Bonferroni correction for multiple testing. As most data had a non-normal distribution or were ranked, correlation was assessed using Spearman’s rho correlation coefficient. Survival data were plotted using the Kaplan–Meier method. Potential clinical factors influencing OS were subjected to univariate analysis using a log-rank test. We report restricted mean OS when median OS was not reached. Due to the low number of events, multivariate analysis using a Cox regression model had to be limited to two estimated hazard ratios. We aimed to evaluate parameters that could be a) objectively evaluated in a retrospective study design and b) be useful in clinical decision-making. Therefore, we decided to test the parameters body mass index (BMI) and Bilsky score as they showed at least a trend in the univariate analysis, but are available to the responsible physician when first evaluating patients for RT. Because it was not always documented and is a somewhat subjective parameter, we decided to not include KPS to reduce bias.

Hazard ratios (HRs) are displayed including their 95% confidence interval (CI). Differences were considered statistically significant for *p* < 0.05. Due to the small sample size we decided to also report trends in differences for *p* < 0.1.

## Results

### Patient demographics

We identified 54 patients who met the inclusion criteria of our study. The median age was 67 years (range 42–82). 54 spinal segments were operated on. KPS before surgery was 60 or lower in 22 patients (22/44; 50%). In 10 patients, presurgical KPS could not be assessed due to the retrospective nature of the study and missing documentation. 15 out of 51 patients (29%) had severe comorbidities resulting in an ACE-27 score of 2 or 3. Conversely, 15 patients (29%) had no relevant comorbidities (ACE-27 score 0). 14 patients (26%) were obese (BMI ≥ 30). Body mass index was significantly higher for patients with ACE-27 scores 2 or 3 (28.7 ± 4.43) as compared to 0 or 1 (25.7 ± 3.39; *p* < 0.01). The median follow-up time was 63 months (95% CI: 49–94 months). Further details are given in Table 1.

**Table 1:** Patient characteristics

Characteristic	*N* = 54^a^
*Age (years)*	67 (59–72)
*Elderly (65 or older)*	
Elderly	32 (59%)
Non-elderly	22 (41%)
*Sex*	
Male	37 (69%)
Female	17 (31%)
*Karnofsky performance score before radiotherapy*	
< 70	12 (23%)
≥ 70	41 (77%)
*rISS stage at diagnosis*	
1	8 (22%)
2	27 (73%)
3	2 (5.4%)
*High-risk aberrations at initial diagnosis*	
No high-risk mutation	37 (93%)
High-risk mutation (del17p)	3 (7.5%)
*Bilsky score*	
0	12 (23%)
1a	8 (15%)
1b	9 (17%)
1c	11 (21%)
2	5 (9.4%)
3	8 (15%)
*SINS at start of radiotherapy*	10.00 (9.00–12.00)
*ACE-27 score before radiotherapy*	
0	15 (29%)
1	21 (41%)
2	10 (20%)
3	5 (9.8%)
*Body mass index*	27.3 (24.4–30.3)
Unknown	2
*Body mass index before radiotherapy*	
BMI 30 or higher	14 (27%)
BMI below 30	38 (73%)
*Smokers*	11 (20%)
*Bisphosphonate therapy*	32 (62%)
Unknown	2

*rISS* revised International Staging System, *SINS* spinal instability neoplastic score, *ACE-27* adult comorbidity evaluation-27 score

^a^Median (IQR); *n* (%)

### Symptoms, disease extension, and spinal scores

The most common indication for surgery was vertebral instability (*n* = 40/54; 74%) followed by neurologic symptoms (*n* = 5/54; 9%). In another 5 cases, surgery was indicated due to both instability and neurological symptoms, while 4 patients received spinal surgery for pain relief (8%).

In nearly all patients, RT was indicated due to the postsurgical setting (*n* = 51/54; 94%). In two patients, RT was also indicated due to instability (4%), while one patient received RT due to persistent pain after surgery (2%). Although pain was not the reason that RT was indicated, 38 patients (70%) reported pain before RT, while only 11 patients (20%) reported some kind of persistent neurologic symptoms, including pain, after surgery (Table 2). Further details regarding treatment modalities are outlined in Table 2.

**Table 2:** Radiotherapy characteristics

Characteristic	*N* = 54^a^
*Total radiation dose*	30.00 (20.00–44.00)
*Alpha/beta* *=* *2* *Gy*	37.50 (26.00–44.00)
*Dose per fraction*	3.00 (2.00–4.00)
*RT scheme*	
20 fractions of 4 Gy	1 (1.9%)
26 fractions of 2 Gy	1 (1.9%)
30 fractions of 2 Gy	6 (11%)
30 fractions of 2.5 Gy	1 (1.9%)
30 fractions of 3 Gy	42 (78%)
40 fractions of 2 Gy	2 (3.7%)
44 fractions of 2 Gy	1 (1.9%)
*Number of vertebrae irradiated*	3.50 (1.00–9.00)
*Number of vertebrae irradiated*	
1	5 (9.3%)
2–4	25 (46%)
5+	24 (44%)
*Location of RT field*	
Cervical spine	4 (7.4%)
Thoracic spine	24 (44%)
Lumbar spine	8 (15%)
Sacral	1 (1.9%)
Cervical and thoracic spine	2 (3.7%)
Thoracic and lumbar spine	11 (20%)
Lumbar and sacral spine	4 (7.4%)
*Surgical procedure*	
Spondylodesis	22 (41%)
Debulking with laminectomy	15 (28%)
Spondylodesis and laminectomy	12 (22%)
Kyphoplasty	5 (9.3%)
*Pain before radiotherapy*	38 (70%)
*Pain after radiotherapy*	
No pain	35 (66%)
Less pain	14 (26%)
Same level of pain	2 (3.8%)
New/more pain	2 (3.8%)

*RT* radiotherapy

^a^Median (range); *n* (%)

Spinal lesions or PTV involved the junctional zone in most patients (*n* = 35/54; 65%). 43 patients (80%) had fractures of varying degrees.

Average SINS score was 10.3 ± 2.5 before RT. Most patients (*n* = 41/54; 76%) had potentially instable lesions, while only 4 patients (7.5%) had stable lesions. Further details regarding spinal scores and stability of bone lesions can be found in Table 1. Most patients had a Bilsky score of 1 (*n* = 28; 53%), while there were nearly equal numbers of patients with a Bilsky score of 0 (*n* = 12; 22%) or at least 2 (*n* = 13; 25%). Bilsky scores did not differ significantly between patients who reported improved or no pain and those with persistent or increased pain after RT (*p* < 0.2).

### Surgery and adjuvant therapies

Surgical procedures used were spondylodesis (*n* = 22, 41%), “tumor debulking” with laminectomy (*n* = 15, 28%), spondylodesis combined with laminectomy (*n* = 12; 22%), or kyphoplasty (*n* = 5; 9%). Of these, 40 (74%) surgeries were performed before or within 30 days of diagnosis. Blood loss as estimated by the anesthesiologist was significantly different between groups defined by surgical procedure (*p* < 0.05). However, pairwise Wilcoxon tests did not show significant differences between two specific groups. In contrast, there were no significant differences in perioperative blood transfusions between groups (*p* = 0.4). The severity of postoperative complications also did not differ significantly between groups (*p* = 0.3). Median time from surgery to discharge was 11.5 days (95% CI 9–16 days), while median time between surgery and start of RT was 34.5 days (95% CI 29–46 days).

Of 54 patients, 22 did not receive systemic therapy prior to RT, while the most common systemic therapy prior to RT consisted of bortezomib plus glucocorticoids, either with or without an additional agent (*n* = 21; 66%). 6 patients (11%) received systemic therapies and RT simultaneously. More details on systemic therapy regimens prior to RT can be seen in Table 3.

**Table 3:** Systemic therapy characteristics

Characteristic	*N* = 54^a^
*Total number of stem cell transplantations*	
0	23 (43%)
1	23 (43%)
2	8 (15%)
*Total number of stem cell transplantations after RT*	
0	29 (54%)
1	20 (37%)
2	5 (9.3%)
*Systemic therapy at any point*	52 (96%)
*Systemic therapy before RT*	35 (65%)
*Systemic therapies before RT*	
Bortezomib/corticosteroids	8 (23%)
Bortezomib/corticosteroids in combination with another agent	13 (37%)
Corticosteroid monotherapy	4 (11%)
Lenalidomide	1 (3%)
Cyclophosphamide, doxorubicin, and corticosteroids	7 (20%)
High-dose melphalan	6 (17%)
Other regimens	8 (23%)
Unknown	1 (3%)
*Simultaneous systemic therapies during RT*	
Carfilzomib	1 (2%)
Bortezomib/corticosteroids	5 (9%)
*Systemic therapy after RT*	51 (94%)

*RT* radiotherapy

^a^*n* (%)

**Table 4:** Adverse events

	Toxicity grade (CTCAE version 05)			
Toxicity	1	2	3+	Total
Dysphagia	3	1	0	4
Nausea	0	4	0	4
Fatigue	1	1	0	2
Emesis	1	0	0	1
Diarrhea	0	0	0	0
Radiodermatitis	1	0	0	1
Leukopenia	0	0	0	0
Esophagitis	0	0	0	0
Xerostomia	0	0	0	0
Dysgeusia	0	0	0	0
*Total*	6	6	0	12

*CTCAE *Common Terminology Criteria for Adverse Events

Each operated spinal segment received postoperative RT with a median total dose of 30 Gy (range 20–44 Gy) in 10 fractions (range 5–22 fractions). In most patients (*n* = 48/54; 89%), more than one vertebra was irradiated. We observed a significantly better prognosis for patients who did not receive a standard RT regimen (either 30 Gy/10 fractions or 20 Gy/5 fractions; *p* < 0.05). These patients received normofractionated RT with doses ranging between 30 and 44 Gy. During the entire disease course, 32 patients received bisphosphonate therapy (*n* = 32/52; 62%).

### Treatment outcomes

The 3‑ and 5‑year overall survival (OS) was 74.9% (95% CI: 63.5–88.4%) and 58% (95% CI: 44.5–75.6%), respectively. Median survival was not reached, 75% survival was 34.3 months (95% CI: 28.7–95.4 months).

Good to adequate presurgical Karnofsky performance scores (KPS ≥ 70) showed a trend toward improved OS (mean OS: 109 vs. 68.2 months; *p* < 0.1), while patients with preradiotherapy KPS ≥ 70 had significantly better OS (median OS: 39.8 months vs. not applicable (NA); *p* < 0.01). McNemar’s test showed significant improvements in KPS after surgery (KPS ≥ 70: 50% vs. 79.2%; *p* < 0.01).

RT showed significant improvements in pain, as 38 patients (71%) reported some degree of pain after surgery and before RT. However, the majority of patients reported no pain (*n* = 35/53; 66%) or decreased pain (*n* = 14/53; 26%) after RT. Only two patients (4%) reported increased pain after RT. Acute toxicities were sparse (*n* = 13/54; 24%) and no higher-grade adverse events (Common Terminology Criteria for Adverse Events (CTCAE) grade 3) occurred (Table 4). There was neither interruption of RT nor delay of the first systemic therapy cycle after RT due to hematological toxicities. We observed no local recurrence within the treatment field.

**Fig. 1:**
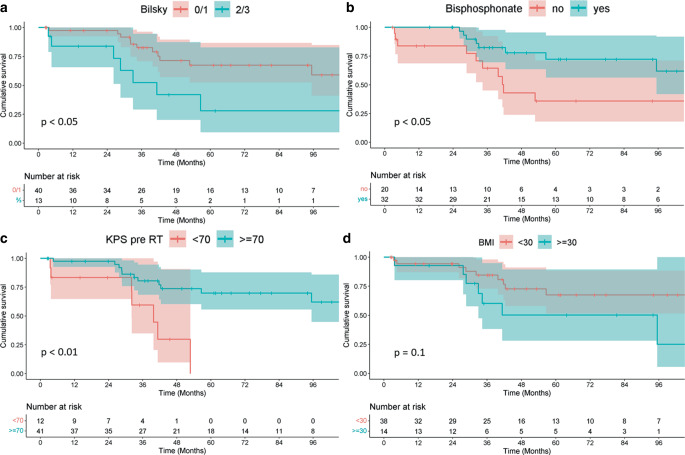
Kaplan–Meier plots for overall survival when stratifying patients for **a** Bilsky score before radiotherapy (*RT*); **b** bisphosphonate therapy; **c** Karnofsky performance score (*KPS*) before RT; **d** body mass index. Bisphosphonate therapy, pre-RT KPS ≥ 70 and low Bilsky scores of 0 or 1 proved to be significant prognostic factors in univariate analysis. Lower BMI does show a trend towards improved overall survival, albeit not statistically significant at *p* < 0.05

While there was no significant difference in OS depending on age (*p* = 0.8), bisphosphonate therapy (mean OS: 108.5 vs. 70.4 months; *p* < 0.05) and a Bilsky score of 0 or 1 (mean OS: 104 vs. 62 months; *p* < 0.05) were associated with significantly better survival. Patients with a body mass index below 30 had a trend towards improved prognosis (mean OS: 107.1 vs. 74.2 months; *p* = 0.1). Corresponding Kaplan–Meier plots and risk tables for factors that showed a trend in survival differences can be seen in Fig. 1. We observed only two SRE (4%).

**Fig. 2:**
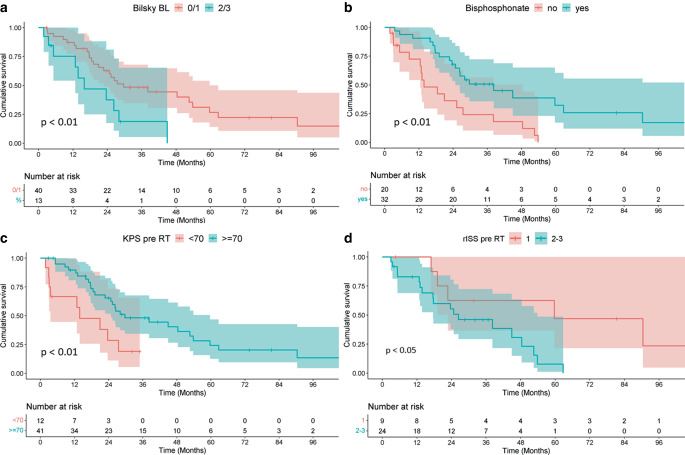
Kaplan–Meier plots for progression-free survival when stratifying patients for **a** Bilsky score at baseline (*BL*) before radiotherapy (*RT*); **b** bisphosphonate therapy; **c** Karnofsky performance score (*KPS*) before RT; **d** revised International Staging System (*rISS*) stage. All abovementioned factors proved to be significant prognostic factors for progression-free survival in univariate analysis

Median progression-free survival was 26.5 months (95% CI: 20.9–48.4 months). KPS before RT (median PFS: 13.8 vs. 29.7 months; *p* < 0.01), revised International Staging System (rISS) stage before RT (median PFS: 26.5 vs. 59.7 months; *p* < 0.05), bisphosphonate therapy (median PFS: 13.8 vs. 38.2 months; *p* < 0.001), and Bilsky score of 0/1 (median PFS: 29.7 vs. 15.9 months; *p* < 0.01) were significantly associated with PFS (Fig. 2).

In the multivariate Cox model for OS, a Bilsky score of 2 or 3 showed a significantly increased hazard ratio (HR 3.89; 95 CI 1.4–10.7; *p* < 0.01). Obesity showed a statistically non-significant trend towards increased HR (HR: 2.57; 95% CI 0.94–7.1; *p* = 0.067).

In the multivariate Cox model for PFS, a Bilsky score of 2 or 3 showed a significantly increased hazard ratio (HR 2.78; 95 CI 1.27–6.1; *p* < 0.05). Obesity showed no sign of an increased HR (HR: 1.16; 95% CI 0.55–2.4; *p* = 0.7).

## Discussion

To our knowledge, this is the first study to demonstrate both the safety and efficacy of RT in MM patients in an exclusively postsurgical setting of spinal lesions. Surgery was able to increase KPS and alleviate symptoms significantly in the short term, preventing long-term neurological symptoms. In addition, RT was safe and able to further alleviate pain and stabilize the postoperative site, as can be seen by the low number of observed SRE (one fracture after RT; one lesion required repeated irradiation), the excellent local control without any in-field recurrences, and the lack of significant side effects (no acute toxicity ≥ grade 3; no late toxicity). While there was no group for comparison, our study strongly supports the current clinical practice of postoperative RT, as we observed no local recurrences in spite of many patients (*n* = 41) developing distant or serologic recurrence at some point. As short-term RT, e.g., 20 Gy in 5 fractions, may be adequate in a more palliative setting, factors that define patients with poor prognosis should be used to guide clinical decision-making during dose prescription [14].

We observed significantly improved OS in patients with a good performance status pre-RT (KPS ≥ 70), in patients who received bisphosphonates, and in patients with a Bilsky score of 0 or 1. We also saw a non-significant trend for increased OS in patients with lower BMI.

We observed similar results for effects on progression-free survival. In addition, the revised International Staging System (rISS) stage pre-RT was also significant. We believe this discrepancy between OS and PFS to be due to the higher number of events in the latter analysis. Additionally, we also observed no effect of BMI on PFS, which did not seem surprising. Multivariate analysis deemed Bilsky score to be significant for both OS and PFS. As we observed no local recurrence, PFS events occurred due to either new distant osteolytic lesions, serological progression, or death. One possible explanation for the association between the Bilsky score as a local evaluation and distant progression of disease might be a more aggressive disease. MM extending beyond the bone might be a sign of a more aggressive phenotype. There is no clinical evidence for this theory and further studies are needed. Some other works on the Bilsky score as a prognostic parameter after spinal surgery already exist, with similar results [24]. However, these works focus on metastatic solid tumors instead of MM.

While we did not focus on this aspect in our analysis, hematological toxicities were comparatively mild. There were no delays of or interruptions to treatment during or shortly after RT due to hematological toxicities. Oertel et al. performed a detailed analysis on hematological toxicities in which they could show that patients receiving systemic therapy and RT simultaneously are at an increased risk for high-grade hematological toxicities [28]. In our cohort, only 6 patients received simultaneous application of systemic therapies and RT, while 53 out of 83 patients in the cohort analyzed by Oertel et al. did. This discrepancy might explain the comparatively good tolerance of RT in our cohort.

As we exclusively evaluated patients with prior surgery to the irradiated region, the cohort is comparatively homogeneous regarding the state of disease at the start of RT.

The results of our study are comparable to other works on the effect of RT on MM lesions [9, 12, 29]. However, while these works focused on a more diverse patient cohort, here we are able to demonstrate similar results exclusively for patients who had received prior surgery. Notably, many patients had a comparatively long time to discharge after surgery. This was explained by the fact that many patients received their diagnosis due to the surgery and were transferred immediately to our hemato-oncology department to start with either systemic therapy or preparation for autologous stem cell transplantation.

Nonetheless, some limitations of this study have to be addressed. First, the retrospective nature of the study has to be mentioned. Some patients received surgery in other hospitals due to the emergency nature of sudden neurological symptoms or severe pain. For these patients, details regarding the surgical procedure were often limited. Additionally, the stage of disease at diagnosis according to the revised International Staging System was missing in 18 patients. To address the retrospective nature of this study, we chose the two factors that could be defined objectively and were independent of current therapy regimens, namely Bilsky score and BMI.

Second, possible confounders such as the nature and extent of systemic therapies might have influenced patient outcome. However, this effect should be comparatively small, since almost all patients received systemic therapy according to internal guidelines of our hemato-oncology department.

Finally, immortal time bias was a theoretical problem. However, due to small time intervals between the last RT application and parameters evaluated after radiation therapy such as pain response, the bias should have been comparatively small. Furthermore, most parameters reported here were assessed before RT and therefore this bias did not exist.

In conclusion, we were also able to show that the current standard of RT after spinal surgery for osteolytic lesions of MM is safe and associated with a low acute and chronic toxicity profile. Furthermore, the low rate of SRE and the absence of in-field recurrences demonstrate the efficacy of RT in MM patients. The Bilsky score is an easy-to-calculate prognostic tool and should be considered before dose prescription, given its prognostic value in MM patients receiving postoperative RT after spinal surgery.

### Supplementary Information

CONSORT diagram of patient selection
