# Supplementary figures and images for: Excellent long-term pain response and local control following postoperative radiotherapy in patients with multiple myeloma
Source: Strahlenther Onkol. 2024 Jan 30;200(7):633–41. doi: 10.1007/s00066-024-02198-7 (PMC11186884; doi:10.1007/s00066-024-02198-7)

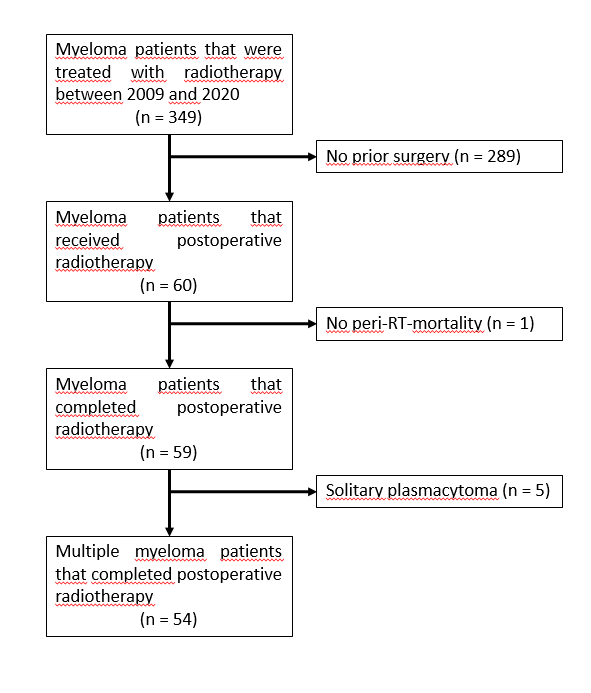

Supplement: Supplementary file 1 — CONSORT diagram of patient selection [file 66_2024_2198_MOESM1_ESM.png]
